# Evaluation of the Potential of Metal–Organic Compounds ZIF-8 and F300 in a Membrane Filtration–Adsorption Process for the Removal of Antibiotics from Water

**DOI:** 10.3390/antibiotics14060619

**Published:** 2025-06-18

**Authors:** Daniel Polak, Szymon Kamocki, Maciej Szwast

**Affiliations:** Faculty of Chemical and Process Engineering, Warsaw University of Technology, Warynskiego 1, 00-645 Warsaw, Poland; daniel.polak@pw.edu.pl (D.P.); szkamocki@gmail.com (S.K.)

**Keywords:** adsorption, membranes, MOF, filtration, tetracycline, sulfadiazine, water

## Abstract

Background/Objectives: Antibiotic contamination in water sources is a growing global concern, contributing to environmental degradation and the proliferation of antimicrobial resistance. Traditional treatment methods, such as advanced oxidation or high-pressure membrane processes, are often energy-intensive and economically unsustainable for large-scale or decentralized applications. This study explores the potential of two cost-effective, commercially available metal–organic frameworks (MOFs), ZIF-8 and F300, to improve the performance of membrane-based filtration–adsorption systems for removing tetracycline and sulfadiazine from water. Methods: Batch adsorption experiments were performed to evaluate the uptake capacities, kinetics, and isotherms of both MOFs toward the selected antibiotics. The membranes were modified using a low-cost silane-assisted deposition of MOF particles and tested in a microfiltration system. Removal efficiencies and water permeability were assessed and kinetic and isotherm models were applied to understand the adsorption mechanisms. Results: ZIF-8 showed superior adsorption performance, with maximum capacities of 442.2 mg/g for tetracycline and 219.3 mg/g for sulfadiazine. F300 was effective only for tetracycline. Membranes modified with ZIF-8 improved pharmaceutical removal by 187% (tetracycline) and 224% (sulfadiazine) compared to unmodified membranes. Although permeability decreased due to increased hydrophobicity, the materials and processes remained economically favorable. Conclusions: This study demonstrates that MOF-modified ceramic membranes, particularly those incorporating ZIF-8, offer a low-cost, scalable, and energy-efficient alternative for pharmaceutical removal from water. The approach combines strong environmental impact with economic viability, making it attractive for broader implementation in water treatment systems.

## 1. Introduction

Water contamination with pharmaceutical waste, including antibiotics, is one of the most serious environmental challenges of the modern world [1]. The presence of pharmaceutical compounds in drinking water and aquatic ecosystems poses significant risks to public health and biological balance, particularly by contributing to bacterial resistance to antibiotics [2,3]. The effective treatment of such wastewater requires the use of advanced separation technologies capable of removing even trace amounts of contaminants [4].

Despite growing concern over pharmaceutical residues in aquatic environments, including antibiotics, there are currently no binding legal limits for their concentrations in water within the European Union. Instead, monitoring relies on the Watch List under the Water Framework Directive, which only recommends surveillance of selected substances [5]. This regulatory gap complicates efforts to assess environmental risk, particularly in the context of antimicrobial resistance (AMR). The absence of legally defined thresholds also makes it difficult to establish clear reference points in the scientific literature, including in this article, limiting the comparability and regulatory relevance of reported data.

Currently available technologies for removing pharmaceutical compounds from water exhibit limited efficiency, particularly in treating water containing trace levels of these pollutants [6,7]. The most commonly applied methods include adsorption on activated carbon [8,9] or other sorbents [10,11], membrane filtration [12,13,14], advanced oxidation processes [15], chlorination [16], bioreactors [17,18] and photocatalysis [19,20].

The most advanced among the aforementioned methods are membrane technologies, which are often considered one of the primary processes for the removal of pharmaceuticals from aquatic environments. Despite the advances in membrane technologies, such as ultrafiltration and nanofiltration, their application in pharmaceutical removal faces notable limitations. These include poor selectivity for small, hydrophilic molecules, membrane fouling, and high energy consumption in pressure-driven systems [13,21]. Moreover, traditional membranes lack the ability to selectively adsorb and retain micropollutants at trace concentrations. Increasing attention is now being given to hybrid methods that combine at least two different purification processes. In this context, metal–organic framework (MOF)-modified membranes offer a promising alternative. MOFs possess tailorable pore structures, large surface areas, and functional groups that enable targeted interactions with antibiotic molecules [22]. When incorporated into membrane matrices, they enhance adsorption selectivity and capacity, effectively transforming the membrane from a purely physical barrier into an active separation material. As such, MOF–membrane composites address the dual challenge of low retention efficiency and costly operation, offering a feasible path toward high-performance, energy-efficient water treatment.

To overcome the limitations of single-method treatment approaches, hybrid or integrated systems combining multiple removal mechanisms are increasingly investigated. These systems often yield synergistic effects, resulting in enhanced contaminant removal efficiency while also offering potential operational flexibility [23,24]. In particular, the integration of adsorption and membrane filtration processes allows simultaneous physical separation and molecular binding, enabling the treatment of both particulate and dissolved contaminants. For instance, studies show that hybrid membrane systems achieve significantly higher contaminant removal rates compared to conventional membranes [25]. The scientific community also concurs that the hybridization of two or more processes into a single operation, particularly when conducted within a single apparatus, results in significant cost savings—both in terms of capital investment and operational energy consumption [23,26]. One such example, discussed in this article, is the combination of the adsorption properties of metal–organic frameworks (MOFs) with the filtration capabilities of membranes.

MOFs are materials with unique properties, characterized by high porosity, large specific surface area, and the possibility of precise chemical functionalization [27,28]. Due to these characteristics, MOFs exhibit excellent adsorption properties, enabling the selective capture of antibiotics and other pharmaceuticals from water. MOFs possess properties that enable selective adsorption of antibiotics through various physicochemical mechanisms [29,30], which are further discussed in Section 3.5. For example, research findings indicate that MOFs such as MIL-101, UiO-66, and ZIF-8 can effectively adsorb antibiotics with efficiencies exceeding 90%, making them some of the most effective materials for pharmaceutical wastewater treatment [29]. The ability to synthesize diverse MOF structures and subsequently functionalize them allows for tailoring their properties to specific contaminants [31]. Table 1 lists the types of antibiotics and the names of the MOF compounds that were tested in the process of their adsorption.

**Table 1:** Antibiotics and MOFs as their adsorbents.

Antibiotic	MOF	Synthesis Method	References
Amoxicillin	MIL-53(Al)	Hydrothermal	[32,33]
Ciprofloxacin	UiO-66-NH2	Solvothermal	[34]
Nalidixic Acid	PCN-224	Solvothermal	[35]
Neomycin	SA-g-P3AP MOF(Fe)/Ag	Solvothermal	[32]
Norfloxacin	UiO-66-NH2	Solvothermal	[34]
Ofloxacin	PCN-224	Solvothermal	[35]
Tetracycline	MIL-101-Fe	Solvothermal	[36,37]
	NH2-MIL-101-Fe	Solvothermal	[36]
	Fe/MIL-100(Fe)	Hyrothermal	[38]
	ZIF-8	Precipitation	[39]
	ZIF-67	Precipitation	[39]
	HKUST-1	Solvothermal	[39]
	Fe-BTC	n/a	[39]
	CoFe	Hyrothermal	[40]
	UiO-66	Hyrothermal	[41,42]
	MOF-5	Solvothermal	[37]
	Cu-ZIF-8	Solvothermal	[37]

The use of MOFs in the form of free adsorbents presents technological limitations. A promising approach to addressing these limitations is the integration of MOFs with membranes, which results in the development of membranes with entirely new properties, particularly improved selectivity. MOF–membrane composites, through the synergistic combination of MOF and membrane properties, enhance separation selectivity, improve water permeability, and reduce membrane fouling. The scientific literature contains numerous studies on the fabrication of membranes incorporating MOFs. These include both thin-film membranes composed entirely of MOFs [43,44] and mixed matrix membranes (MMMs), where MOF particles are embedded in a polymer matrix [45,46]. The diversity of MOFs used and the antibiotics they adsorb is substantial.

The objective of this study was to evaluate the feasibility of using metal–organic frameworks ZIF-8 and F300 in a membrane-based filtration–adsorption process for the removal of pharmaceutical compounds from water. Additionally, the adsorption properties of the modified membranes were investigated. The results demonstrated that the efficiency of the adsorption process depends on the physicochemical properties of both the MOF particles and the pharmaceutical compounds. Membranes containing ZIF-8 exhibited a higher sorption capacity for both tetracycline and sulfadiazine.

We selected two types of antibiotics, namely tetracycline and sulfadiazine. Both compounds belong to classes of antibiotics (tetracyclines and sulfonamides, respectively) that are extensively applied in human medicine, veterinary practices, and livestock farming. Their frequent usage, particularly in animal husbandry, leads to significant emissions into the environment through wastewater discharge and agricultural runoff [47,48]. Among pharmaceutical contaminants, tetracycline and sulfadiazine are particularly concerning due to their widespread use and frequent detection in surface water, groundwater, and even drinking water [49,50,51]. These compounds are known to persist in the environment, contribute to the development of antibiotic resistance genes (ARGs), and interfere with aquatic ecosystems, posing long-term risks to both human and environmental health [52,53]. Tetracyclines have remained a cornerstone of antimicrobial therapy for decades due to their broad-spectrum activity against Gram-positive, Gram-negative, and atypical bacteria. Despite the introduction of new antibiotic classes, their clinical use has not diminished. The development of third-generation derivatives—such as tigecycline, eravacycline, and omadacycline—has further enhanced their efficacy, including against multidrug-resistant strains [54,55]. Moreover, emerging evidence suggests that these novel tetracyclines may possess bactericidal, anti-inflammatory, and anticancer properties, making them promising agents beyond traditional infectious disease indications. Sulfadiazine, in turn, is widely used in veterinary medicine and remains one of the fundamental drugs in the treatment of toxoplasmosis [56]. It is also still employed in human clinical practice [57]. Increasing attention has been given to the application of this compound in combination with silver ions, resulting in the formation of silver sulfadiazine [58,59]. This formulation is currently utilized in the treatment of burns [60] as well as in dermocosmetic applications for managing skin reactions in patients undergoing oncological radiotherapy [61].

While numerous studies have demonstrated the potential of MOF materials for the adsorption of antibiotics from aqueous environments, the practical integration of these materials into membrane systems remains a significant challenge. The fabrication of MOF-containing membranes that not only retain the high adsorption capacity of the MOFs but also provide stable, efficient, and scalable filtration performance is still under development. Embedding MOFs into polymeric matrices or on membrane surfaces often results in reduced accessibility of active sites, agglomeration, or compromised membrane integrity. In this study, two commercially available MOF structures were employed: ZIF-8 and F300. Both materials exhibit excellent adsorption properties toward pharmaceutical compounds, dyes, gases and metal ions [62,63,64,65,66]. Furthermore, they possess characteristics desirable for membrane applications, such as antibacterial [67,68] and antifungal activity [69], which can contribute to the reduction of membrane biofouling. ZIF-8 and F300 are characterized by chemical and mechanical durability [70,71,72], which allows the use of various methods of membrane modification and conducting the filtration process under different process conditions. Therefore, it is critical to explore optimized strategies for incorporating MOFs into membrane architectures, focusing on achieving synergistic effects between adsorption and separation. This study addresses this gap by evaluating hybrid membranes containing ZIF-8 and F300 for effective antibiotic removal, aiming to bridge the gap between fundamental MOF sorption studies and functional membrane technologies suitable for real-world water treatment applications.

## 2. Materials and Methods

### 2.1. Materials

In the presented research, the commercial metal–organic frameworks ZIF-8 (Sigma-Aldrich, Saint Louis, MO, USA) and F300 (Sigma-Aldrich, Saint Louis, MO, USA) were used. Commercially available nineteen-channel ceramic membranes (Star-Sep, Mantec Filtration, Longton, UK) were used for the tests. These membranes are made of α-Al_2_O_3_, with an average pore diameter of 0.35 µm. The membranes were modified with a solution of octadecyltrichlorosilane (ODTS, Sigma-Aldrich, Saint Louis, MO, USA) in isopropyl alcohol (POL-AURA, Warsaw, Poland). The aqueous solutions of the antibiotics tetracycline (Sigma-Aldrich, Saint Louis, MO, USA) and sulfadiazine (POL-AURA, Warsaw, Poland) were used for adsorption studies. Additionally, a PVDF membrane (Durapore, Bionovo, Legnica, Poland), with a pore diameter of 0.1 µm, was used in a vacuum filtration system. In the research, reverse osmosis water was used.

### 2.2. Testing of MOF Adsorption Properties

In order to determine the adsorption properties of ZIF-8 and F300, tests were carried out under different conditions. The tests were conducted in a batch system for various MOF masses, different pharmaceutical substance concentrations, and varying adsorption times. In this method, aqueous solutions of pharmaceuticals were mixed with MOF particles, and after a predetermined time, MOF particles were separated from the pharmaceutical solution using a vacuum filtration system. The solution volume was 50 mL. The concentration of the pharmaceutical substance was determined using UV–Vis spectroscopy (Genesys 10S UV–Vis, Thermo Fisher Scientific, Waltham, MA, USA).

In the first part of the research, the impact of MOF concentration on pharmaceutical adsorption was tested. To achieve this, the adsorbed mass of pharmaceuticals for three different MOF masses (different MOF solution concentrations), 0.05 mg, 0.0125 mg, and 0.0025 mg, was determined. At lower concentrations, no significant adsorption effects were observed, while at higher concentrations, particle agglomeration occurred, making further investigation challenging. The concentration of pharmaceuticals was measured after 90 min. The initial pharmaceutical concentration was 40 mg/dm^3^, and the adsorbed pharmaceutical mass on MOF was calculated using Equation (1):

(1) $$q_{t}=\frac{\left(c_{0}-c_{t}\right)}{m_{MOF}}\cdot V_{BS}$$

where: *q_t_* [mg/g] is the mass of adsorbed pharmaceutical, *c*_0_ [mg/dm^3^] and *c_t_* [mg/dm^3^] is the initial pharmaceutical concentration and the concentration after time *t* [min], *V_BS_* [dm^3^] = 50 cm^3^ is the solution volume and *m_MOF_* [g] is the MOF mass.

In the second part of the research, the kinetic model of pharmaceutical adsorption on the MOF surface was determined. To achieve this, the adsorbed pharmaceutical mass was measured at 1 min, 5 min, 10 min, 15 min, 30 min, 60 min, and 90 min. The initial pharmaceutical concentration was 40 mg/dm^3^, and the MOF mass used was 0.0025 mg. To explain the mechanism of tetracycline and sulfadiazine adsorption on the MOF surface, the pseudo-first-order kinetic model (Equation (2)) and pseudo-second-order kinetic model (Equation (3)), Elovich model (Equation (4)) and Intraparticle Diffusion model (IPD) (Equation (5)) were analyzed. These models are most used to describe the adsorption kinetics of pharmaceutical substances on solid surfaces [73,74,75,76].

Pseudo-first-order kinetics:

(2) $$q_{t}=q_{e}\left(1-e^{-k_{1}t}\right)$$

Pseudo-second-order kinetics:

(3) $$q_{t}=qek2tqe+k2tq_{t}=\frac{q_{e}k_{2}t}{q_{e}+k_{2}t}$$

Elovich model:

(4) $$q_{t}=\frac{1}{\beta }\ln\left(1+\alpha \beta t\right)$$

Intraparticle Diffusion model (IPD):

(5) $$q_{t}=k_{d}\sqrt{t}+C$$

where *k*_1_ [1/min], *k*_2_ [mg/(g·min)] and *k_d_* [g/(mg·min^0.5^] are the apparent rate constant, *t* [min] is the process time, *q_e_* (mg/g) is the equilibrium concentration of the adsorbate on the surface, *α* is initial sorption rate in [mg/(g·min)], *β* is activation energy for chemisorption (g/mg) and *C* [mg/g] is a constant depended on thickness and the boundary layer.

In the third part of the research, to further analyze the mechanism of pharmaceutical adsorption on the MOF surface, different adsorption isotherms were tested. For this purpose, the adsorbed pharmaceutical mass was determined for various initial concentrations, i.e., 5 mg/dm^3^, 10 mg/dm^3^, 15 mg/dm^3^, 20 mg/dm^3^, 40 mg/dm^3^, 70 mg/dm^3^, and 120 mg/dm^3^ (only for tetracycline). The pharmaceutical concentration was measured after 90 min of adsorbing process. In the present study, the Freundlich (Equation (6)), Langmuir (Equation (7)), Redlich–Peterson (Equation (8)) and Sips (Equation (9)) models were examined [77,78,79].

Freundlich isotherm:

(6) $$q_{e}=k_{F}{c_{e}}^{\frac{1}{n}}$$

Langmuir isotherm:

(7) $$q_{e}=\frac{k_{L}c_{e}q_{max}}{1+{k_{L}c}_{e}}$$

Redlich-Peterson isotherm:

(8) $$q_{e}=\frac{k_{R}c_{e}}{1+Bc_{e}\beta }$$

Sips isotherm:

(9) $$q_{e}=\frac{q_{max}{\left(k_{L}c_{e}\right)}^{\frac{1}{n}}}{1+{\left({k_{L}c}_{e}\right)}^{\frac{1}{n}}}$$

where *q_e_* [mg/g] is the equilibrium concentration of the adsorbate on the surface, *c_e_* [mg/dm^3^] is the equilibrium concentration of the adsorbate in the liquid phase, and, according to:

- *k_F_* [(mg^(1−(1/n))^·(dm^3^)^1/n^)/g] is an empirical constant representing the adsorption capacity of the material, 1/*n* [−] is a parameter that indicates adsorption intensity
- *k_L_* [dm^3^/g] is the Langmuir constant and *q_max_* [dm^3^/mg] is the theoretical maximum adsorption capacity
- *k_R_* [dm^3^/g] is the Redlich–Peterson constant, *B* [dm^3^/mg] is also constant, *β* [−] is a parameter between 0 and 1. If *β* = 1, the equation reduces to the Langmuir expression.

In the final part of the study, the effect of pH in aqueous solutions of tetracycline and sulfadiazine on the efficiency of the adsorption process was determined. For this purpose, aqueous antibiotic solutions were prepared at pH values of 2.5, 3.5, 5, 6.5, 7.2, 8, and 10.5. Based on the tests conducted, the amount of tetracycline and sulfadiazine adsorbed onto the adsorbent surface was determined. The experiments were carried out at an antibiotic concentration of 40 mg/dm^3^.

### 2.3. Membrane Modification

The chemical modification method was used to coat membrane surfaces with MOF particles. In this method, the octadecyltrichlorosilane (ODTS) anchoring compound forms a connection between MOF particles and the membrane surface. Membrane surfaces were modified via ODTS-assisted deposition of MOF particles using ultrasound-assisted immersion and subsequent washing, following standard protocols [80,81]. The mass concentration of the MOF solution was 0.025%.

### 2.4. Testing Membranes

To evaluate the adsorption properties of the modified membranes, a microfiltration process was conducted. The study was performed on four types of membranes: an unmodified membrane (Unmod), a membrane modified solely with ODTS (Mod-ODTS), and membranes modified with MOF particles, specifically Mod-ZIF-8 and Mod-F300. Based on the adsorption–filtration process, the change in pharmaceutical substance concentration during filtration and the removed mass of pharmaceutical substances per unit membrane filtration area from the feed stream were determined (Equation (10)). The microfiltration test was conducted for 120 min under a feed pressure of 0.5 bar (500 kPa). The initial feed concentration was 40 mg/dm^3^, with a total feed volume of 0.5 dm^3^. The membrane length was 110 mm, and the filtration area was 0.011 m^2^. The experiments were carried out in a closed system with recirculation of both the retentate and permeate streams. Additionally, during the adsorption–filtration process, water permeability was determined based on the permeate volumetric flow rate (Equation (11)).

(10) $$q_{flow}=\frac{\left(c_{0}-c_{t}\right)}{A_{mem}}\cdot V_{FS}$$

where *q_flow_* [mg/g] represents the mass of the removed pharmaceutical compound, *c*_0_ [mg/dm^3^] and *c_t_* [mg/dm^3^] denote the initial concentration of the pharmaceutical and its concentration at a given process time, *V_FS_* [dm^3^] is the solution volume and *A_mem_* [m^2^] is the membrane filtration area.

(11) $$WP=\frac{Q_{p}}{A_{mem}\cdot p_{N}}$$

where *WP* [m^3^/(m^2^·Pa·s] represents the membrane water permeability, *Q_p_* [m^3^/s] is permeate volumetric flow rate, and *p_N_* [Pa] denotes the feed pressure.

Additionally, to determine the influence of the presence of salt ions, a study was conducted using tap water to which 40 mg/dm^3^ of tetracycline or 40 mg/dm^3^ of sulfadiazine was added. The conductivity and pH of the tap water were 720 µS/cm and 6.8, respectively. The composition of the used tap water is: 74.0 mg Na^+^/L, 14.6 mg Mg^2^^+^/L, 71.1 mg Ca^2^^+^/L and 0.07 mg Fe^2+^/L.

## 3. Results and Discussion

The following sections present the results of the research conducted. Firstly, the adsorption properties of MOFs were evaluated by analyzing the mass of pharmaceutical substances adsorbed at different concentrations of MOF particles. Next, the adsorption kinetics were studied using pseudo-first-order, pseudo-second-order, Elovich and IPD models to determine the mechanism of the process. The subsequent step involved the analysis of adsorption isotherms using the Freundlich, Langmuir, Redlich–Peterson and Sips models to characterize the interactions between pharmaceutical substances and the MOF surface. After obtaining the results for MOF powders, the surface modification of ceramic membranes with these particles was carried out. The results demonstrated successful modification are presented, followed by an evaluation of the impact of MOF particle modification on membrane permeability and the effectiveness of pharmaceutical substance removal from the solution.

### 3.1. Evaluation of MOF Adsorption Properties

**Figure 1:**
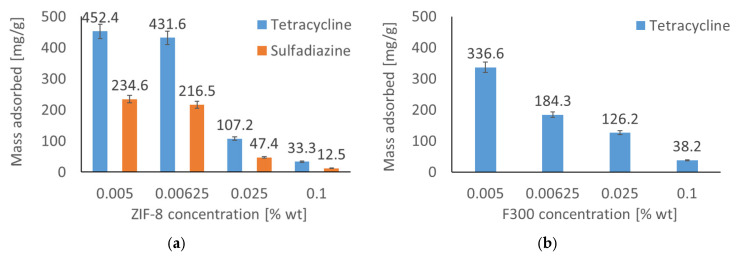
Adsorbed mass of antibiotics (tetracycline and sulfadiazine) as a function of MOF concentration: (**a**) ZIF-8, (**b**) F300. A decrease in adsorption at higher MOF concentrations is observed due to particle agglomeration, which reduces active surface area. ZIF-8 adsorbs both drugs effectively, while F300 is selective for tetracycline.

Figure 1 illustrates the adsorbed mass of the pharmaceutical substance from an aqueous solution as a function of MOF particle concentration. The data indicate an inverse relationship between MOF particle concentration and adsorbed mass; specifically, an increase in MOF particle concentration leads to a decrease in adsorbed substance. This observation can be attributed to particle agglomeration, which results in a reduction of the adsorbent’s effective surface area. This trend was consistent across all drug–MOF systems investigated. ZIF-8 demonstrated superior adsorption performance for both pharmaceutical substances. Conversely, F300 exhibited significant adsorption capacity only towards tetracycline, with the adsorbed mass of sulfadiazine being statistically insignificant. This phenomenon is related both to the pH of the solution used in the study and to the surface properties of the F300 material, which will be discussed in more detail in the following paragraph. Consequently, the sulfadiazine–F300 system was excluded from subsequent analyses. To elucidate the mechanisms underlying the differential adsorption behavior of pharmaceutical substances on the various MOF structures, adsorption kinetics and isotherms were subsequently investigated.

### 3.2. Adsorption Kinetic

**Figure 2:**
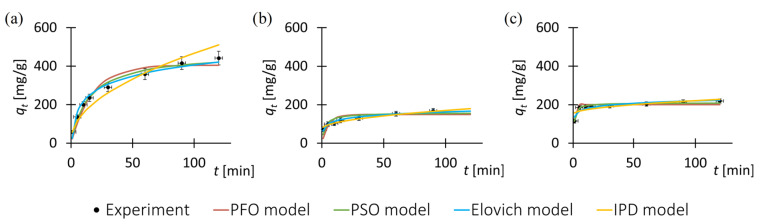
Adsorption kinetics for tested drug–MOF systems: (**a**) tetracycline on ZIF-8, (**b**) tetracycline on F300, (**c**) sulfadiazine on ZIF-8. Experimental data were fitted to kinetic models; complex adsorption mechanism is most probable.

To investigate the adsorption kinetics of the pharmaceutical substances onto the MOF surfaces, experimental data of adsorbed mass at various time intervals were utilized. Figure 2 displays the experimental data alongside the fitted kinetic models, while Table 2 provides a compilation of the determined kinetic model parameters and the corresponding model performance indicators. Each data point in Figure 2 represents the mean of three independent measurements. Error bars indicate the standard error (*SE*) calculated from these replicates. For all data points, the standard error did not exceed 8% of the corresponding mean value.

The adsorption kinetic curves for the antibiotics (Figure 2) exhibit similar trends to those commonly reported in the literature for other classes of MOF materials [82]. This consistency enhances the reliability and scientific validity of the results presented in this study.

As shown in Table 2, all the tested models exhibit a high level of statistical significance (*p* > 0.05), allowing for further analysis. Two indicators were used to evaluate the quality of the model fitting to the experimental data: the sum of squared errors (*SSE*) and the coefficient of determination (*R*^2^). While *SSE* reflects the total prediction error of a model, *R*^2^ provides a more intuitive and universally interpretable measure, particularly useful when comparing different models or analyzing data from different experimental sets. For this reason, further discussion focuses primarily on the *R*^2^ values. It should be noted, however, that with only a few exceptions, the *SEE* values obtained for the individual model fits are of the same order of magnitude.

Based on the obtained experimental data of the adsorbed mass of the pharmaceutical substance during the process, it is observed that the functional trends describing the changes in adsorbed mass are analogous across all drug–MOF systems. However, in the case of tetracycline adsorption on ZIF-8, the adsorption equilibrium state is achieved later compared to the other tested drug–MOF systems. This effect may be attributed to the larger active surface area of the ZIF-8 structure (1300–1800 m^2^/g, producer specification sheet) relative to F300 (120–190 m^2^/g, producer specification sheet), and consequently, a greater number of adsorption sites available for tetracycline molecules. Furthermore, the lower adsorbed mass of sulfadiazine compared to tetracycline, as well as the inability of F300 to adsorb sulfadiazine, may be due to specific chemical interactions between the MOF particles and the pharmaceutical substance molecules.

Analysis of the *R*^2^ values indicates that all the evaluated kinetic models provide a reasonably good fit to the experimental data. However, the pseudo-first-order model demonstrates the weakest performance among them. The remaining models exhibit similarly high coefficients of determination, suggesting comparable predictive capabilities.

The kinetic analysis of the drug adsorption process onto the porous solid revealed that the pseudo-first-order model provides the poorest fit to the experimental data, suggesting that simple physisorption is not the dominant mechanism [83]. In contrast, the pseudo-second-order model yielded *R*^2^ values are high, indicating that the adsorption is likely governed by chemisorption processes involving electron exchange or stronger interactions between the adsorbate and the adsorbent surface [84]. The good fit of the Elovich model further supports the involvement of heterogeneous surface interactions typically associated with chemisorption [85]. Moreover, the intraparticle diffusion model also showed satisfactory agreement with the data, implying that diffusion within the pores of the adsorbent may contribute to the overall rate-limiting step [86]. However, since the plot of *q*_*t*_(_t_) does not pass through the origin, it can be concluded that intraparticle diffusion is not the sole rate-limiting mechanism [86,87]. Overall, the results suggest a complex adsorption process involving both surface interactions and internal diffusion effects.

It is also noteworthy that sulfadiazine adsorption on ZIF-8 particles exhibits rapid kinetics in the initial stages, with a relatively quick attainment of adsorption equilibrium. This phenomenon may be attributed to the intensive sorption of sulfadiazine on the ZIF-8 surface and hindered diffusion of the adsorbate into the pore interiors [88].

### 3.3. Adsorption Isotherms

**Figure 3:**
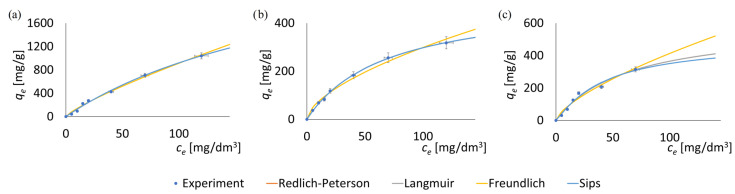
Adsorption isotherms of antibiotics on MOF particles: (**a**) tetracycline on ZIF-8, (**b**) sulfadiazine on ZIF-8, (**c**) tetracycline on F300. Models fitting suggests a predominance of monolayer adsorption.

To investigate the adsorption isotherms, experimental data of the adsorbed mass of pharmaceutical substances at varying initial concentrations were utilized. The experimental results and the fitted adsorption isotherms are presented in Figure 3, while the determined isotherm parameters are summarized in Table 3.

Each data point in Figure 3 represents the mean of three independent measurements. Error bars indicate the standard error (*SE*) calculated from these replicates. For all data points, the standard error did not exceed 9% of the corresponding mean value.

The adsorption isotherms for the antibiotics (Figure 3) exhibit trends consistent with those commonly reported in the literature for other classes of MOF materials [82]. This consistency further confirms the reliability and scientific validity of the results presented in this study.

The two-parameter Freundlich and Langmuir isotherms, along with the three-parameter Redlich–Peterson isotherm and Sips isotherms, were employed to model the adsorption of tetracycline and sulfadiazine onto the surfaces of ZIF-8 and F300. Based on the obtained *R*^2^ values, the Redlich–Peterson isotherm and Langmuir isotherm demonstrated the best fit with the experimental data. The very good fit of the Redlich–Peterson model suggests that the adsorption process does not occur with ideal monolayer formation on the solid surface [89,90]. Very high and high, respectively, *R*^2^ values (>0.97) for the Langmuir and Freundlich models also indicate that the adsorption process in the studied drug–MOF systems involves multiple mechanisms. The presence of these multiple adsorption mechanisms is attributed to the heterogeneous surface of the MOF particles, which contains diverse functional groups [22,91].

As shown in Table 3, all the tested models exhibit a high level of statistical significance (*p* > 0.05), allowing for further analysis. Once more two indicators, namely SSE and *R*^2^, were used to evaluate the quality of the model fitting to the experimental data. In this case, the SSE values are of similar magnitude for all three models, with the Langmuir isotherm exhibiting noticeably lower values. However, all the considered isotherm models show relatively high coefficients of determination (*R*^2^), making it difficult to unequivocally determine which model best represents the adsorption process. These observations suggest that the adsorption process may involve a combination of mechanisms, including monolayer adsorption on relatively homogeneous sites (Langmuir model) [92] as well as adsorption on heterogeneous surfaces with varying affinities (Freundlich model) [93]. Nonetheless, the markedly lower *SEE* values obtained for the Langmuir model suggest a predominance of monolayer adsorption, potentially extending into the internal surfaces of the porous structures without apparent transport limitations.

### 3.4. Effect of pH

**Figure 4:**
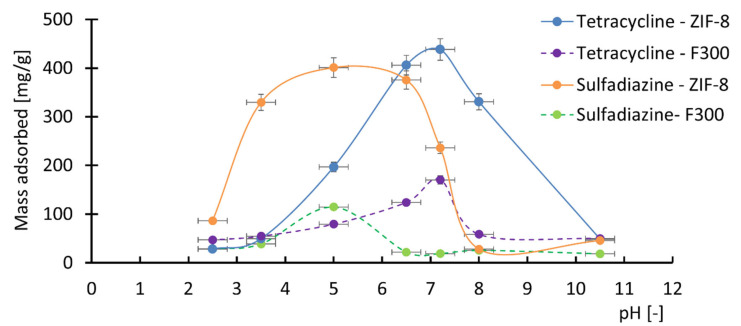
Effect of pH on the adsorbed mass of tetracycline and sulfadiazine on ZIF-8 and F300. The adsorption of tetracycline was most efficient in the pH range of 6–8, while sulfadiazine showed the highest adsorption at pH 4–6. The colored lines presented in the figure do not possess physical significance; they serve solely to illustrate the trends of change.

An important parameter affecting the adsorption of pharmaceutical compounds on MOF surfaces is the pH of the solution. Therefore, adsorption experiments were conducted for tetracycline and sulfadiazine in aqueous solutions with varying pH values. The amounts of adsorbed tetracycline and sulfadiazine on ZIF-8 and F300 at pH 2.5, 3.5, 5, 6.5, 7.2, 8, and 10.5 are presented in Figure 4.

Based on the obtained results (Figure 4), it can be concluded that the pH of the solution significantly influences the amount of pharmaceutical compounds adsorbed onto the surface of MOF structures. This is attributed to changes in the zeta potential of ZIF-8 and F300 [94,95], as well as the pH-dependent ionic forms of tetracycline and sulfadiazine [96,97].

The data indicate that the adsorption of tetracycline is most efficient at pH values between 6 and 8. In this pH range, both ZIF-8 and F300 exhibit negatively charged surfaces, while tetracycline molecules contain both positively and negatively charged moieties (zwitterionic forms). The presence of positively charged groups enables electrostatic attraction with the negatively charged surfaces of ZIF-8 and F300, enhancing adsorption efficiency. In contrast, under acidic (pH < 6) and alkaline (pH > 8) conditions, the adsorption of tetracycline is significantly reduced. In acidic media, the negative surface charge of ZIF-8 and F300 decreases, and at pH values below 3.5, both materials become positively charged. Simultaneously, the proportion of cationic tetracycline species increases, resulting in electrostatic repulsion. In alkaline environments (pH > 8), ZIF-8 and F300 maintain a negative surface charge, while tetracycline shifts toward anionic forms, again leading to repulsion.

Regarding sulfadiazine, the highest adsorption capacity on ZIF-8 and F300 is observed at pH values between 4 and 6. In this range, sulfadiazine predominantly exists in a neutral, non-ionized form, favoring intermolecular interactions with the adsorbent surface. At pH values below 4 and above 6, cationic and anionic forms of sulfadiazine become dominant, respectively, which—similar to tetracycline—leads to electrostatic repulsion and reduced adsorption efficiency.

### 3.5. Analysis of Adsorption Data

Based on the obtained experimental data concerning adsorbed mass, kinetic models, and adsorption isotherms, it is evident that multiple factors contribute to the efficiency of the adsorption process. One significant factor is the agglomeration of MOF particles, which can substantially reduce adsorption efficiency. Another key factor is the specific surface area of the MOF particles and the associated density of adsorption sites, which directly influences the sorption capacity of the material. Furthermore, the adsorption of pharmaceutical substances on MOFs is governed by chemical interactions between the adsorbate and adsorbent. These interactions are contingent upon the chemical functionalities present in both the pharmaceutical substance and the MOF surface. Potential interactions include π–π stacking, electrostatic interactions, acid–base interactions, hydrophobic interactions, hydrogen bonding, and even covalent bonding, all of which may contribute to the binding of pharmaceutical substances to the MOF surface. Additionally, the adsorption process can be limited by single or multiple interacting forces [98]. In the context of pharmaceutical adsorption on MOF surfaces, electrostatic interactions, which are dependent on the zeta potential of the MOF surface and the ionic speciation of the pharmaceutical, are commonly rate-determining. Additionally, the presence of -NH_2_ groups on ZIF-8 and -COOH groups on F300 allows for the formation of hydrogen bonds with the -OH and -NH_2_ groups of tetracycline and the -NH_2_ groups of sulfadiazine. Moreover, π-π stacking interactions can occur between aromatic moieties present in the ZIF-8 structures and the pharmaceutical substances. Additionally, coordination bonds may form between the -OH and -NH_2_ functional groups present in tetracycline and the -NH_2_ group in sulfadiazine, and the Zn^2+^ and Fe^3+^ ions found in the structures of ZIF-8 and F300, respectively. However, it should be emphasized that the occurrence of these interactions between antibiotic molecules and MOF structures is strongly pH dependent. Therefore, pH is a key factor significantly influencing the adsorption of antibiotics onto the surfaces of ZIF-8 and F300. For tetracycline and sulfadiazine, the highest adsorption efficiencies were observed in the pH ranges of 6–8 and 4–6, respectively. Outside these ranges, the amount of adsorbed compound decreased markedly in both cases.

The results also indicate that F300 exhibits a lower adsorption capacity compared to ZIF-8. This can be attributed to the higher specific surface area of ZIF-8 and the presence of imidazolate groups, which are capable of forming π–π stacking interactions with the aromatic rings of the antibiotic molecules. Furthermore, the Fe^3^^+^ ion possesses a higher charge density than Zn^2+^ [99], resulting in a stronger affinity for water molecules. As a consequence, the coordination sites of Fe^3+^ are often occupied by water, reducing their availability as active adsorption centers and thereby limiting the binding of tetracycline and sulfadiazine in the case of F300. In summary, the adsorption of tetracycline and sulfadiazine on ZIF-8 and F300 is governed by a complex interplay of multiple interactions. The involvement of diverse interactions in the adsorption of pharmaceutical substances on MOFs is well-documented in the literature [82,100,101,102].

### 3.6. Membranes’ Properties

**Figure 5:**
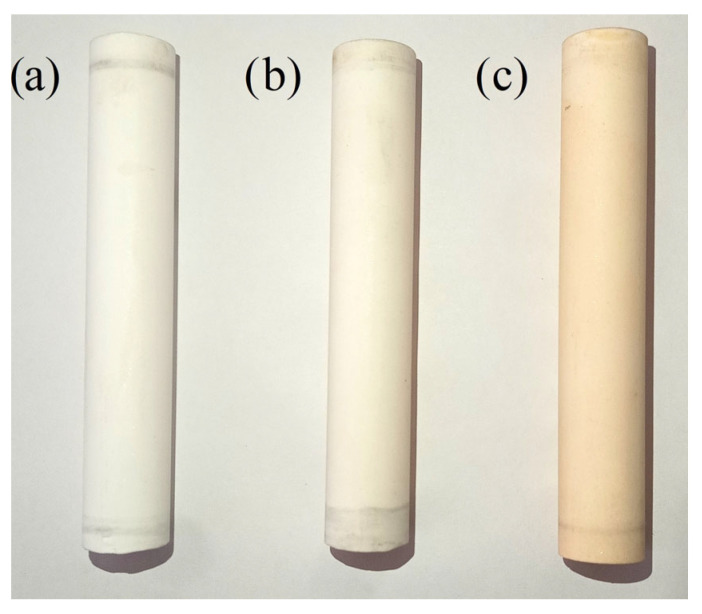
Photo of tested membranes: (**a**) unmodified membrane (Unmod), (**b**) membrane modified by ZIF-8 (Mod-ZIF-8), (**c**) membrane modified by F300 (Mod-F300).

In the study of the integrated adsorption–membrane process, three types of membranes were utilized: an unmodified ceramic membrane (Unmod) and two membranes modified according to the procedure described in Section 2.3—namely, a ceramic membrane with ZIF-8 nanoparticles (Mod-ZIF-8) and a ceramic membrane with F300 nanoparticles (Mod-F300). As a result of the modification process, the obtained membranes are depicted in Figure 5. The presented micrographs reveal differences in the surface morphology of the modified membranes compared to the unmodified membrane. Membranes coated with ZIF-8 particles exhibit a slightly creamy coloration, whereas those coated with F300 particles display an intense orange hue. The observed visual changes are stable, and the membranes retain their appearance even after prolonged operation under working conditions.

**Figure 6:**
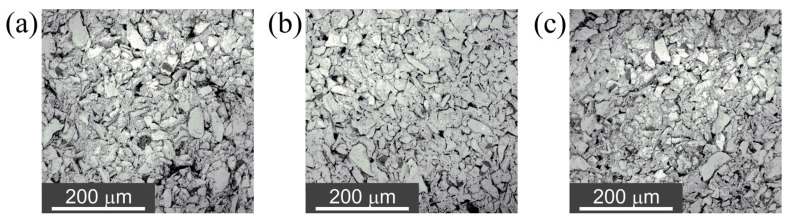
SEM images of tested membranes: (**a**) unmodified membrane (Unmod), (**b**) membrane modified by ZIF-8 (Mod-ZIF-8), (**c**) membrane modified by F300 (Mod-F300).

To investigate micro-scale changes in membrane structure, scanning electron microscopy (SEM) was employed. Special holders were used to allow for imaging of the membrane surfaces without the need for conductive coating. Observations of SEM images for the unmodified membrane and the two modified membranes (Figure 6) indicate that this type of analysis does not yield new information. The coatings formed on the membranes are not detectable with this technique. However, it is evident that the applied modifications did not adversely affect the overall structure of the membranes.

**Figure 7:**
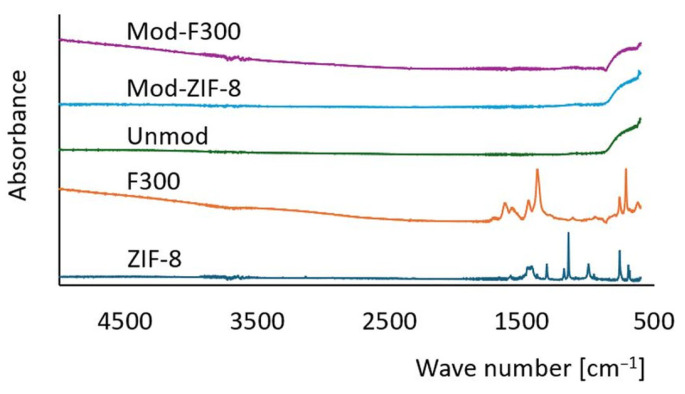
FT-IR spectra of membranes and MOFs. Modified membranes show slight shifts and signal changes compared to the unmodified membrane, indicating MOF presence. A drift in baseline for Mod-F300 supports Fe-based compound integration.

To confirm the presence of nanoparticles on the membrane surface, FT-IR measurements were conducted using the ATR technique (Nicolet iS10, Thermo Scientific, Waltham, MA, USA). The recorded spectra for ZIF-8 and F300 particles, as well as for unmodified and modified membranes, are presented in Figure 7. FT-IR spectra confirmed the presence of MOF structures on the membrane surface, as evidenced by minor spectral shifts and baseline changes. The spectrum of the unmodified membrane demonstrates a typical profile for ceramic materials [103]. From the perspective of assessing the effectiveness of the modification, the analysis of the modified membrane spectra is crucial. It is worth noting that the mass fraction of nanoparticles relative to the membrane mass is small, which may result in the signal from the nanoparticles being overshadowed by the intense signal of the ceramic material [104]. In the case of Mod-ZIF-8 membranes, the recorded spectrum shows only subtle differences compared to the unmodified membrane spectrum, making it challenging to conclusively confirm the effectiveness of the modification. In contrast, for Mod-F300 membranes, despite the absence of distinct characteristic peaks for F300, a baseline drift typical for metal–organic compounds containing Fe atoms [105] was observed, which can be considered evidence of a successful modification.

To further analyze changes in membrane surface properties, contact angle measurements were performed (OCA 25, DataPhysics, Filderstadt, Germany). The measurement results are summarized in Table 4. The obtained data clearly indicate the effectiveness of the applied modifications. The unmodified ceramic membrane exhibits a strongly hydrophilic nature, whereas ODTS modification leads to its hydrophobization [106]. The addition of nanoparticles, particularly the superhydrophobic ZIF-8 particles [107] and the hydrophobic F300 particles [108], further enhances the hydrophobicity of the membrane surfaces. The obtained results confirm the effectiveness of the applied modification procedures.

**Figure 8:**
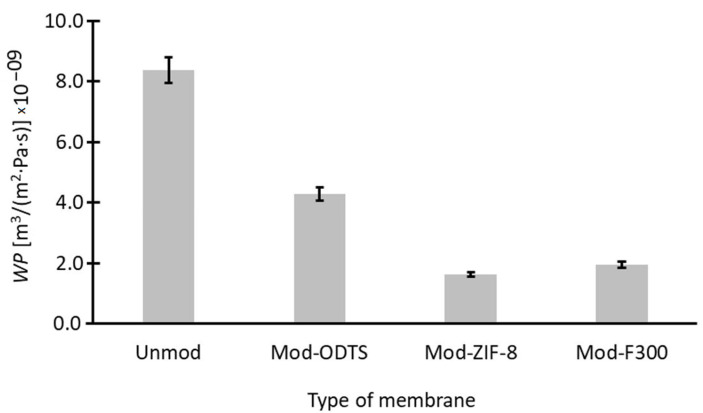
Water permeability of membranes. Hydrophobization through ODTS and MOF coating leads to reduced permeability, with Mod-ZIF-8 showing the lowest value, correlating with its highest contact angle.

The final stage of the modified membrane analysis involved assessing their water permeability (*WP*). The measurement results and calculations performed according to Equation (11) are presented in Figure 8. The initially relatively high *WP* value for the unmodified membrane decreases with each modification stage. In the case of ODTS modification, an approximately twofold reduction in *WP* is observed, while additional modification with nanoparticles further decreases water permeability. Nevertheless, the water permeability values of the modified membranes still exhibit acceptable magnitudes for industrial applications and demonstrate performance comparable to or even exceeding that reported in other pieces of literature [109,110]. The observed reduction in water flux following surface modification is primarily attributed to the increased hydrophobicity of the membrane surface, as confirmed by contact angle measurements (Table 4). Notably, water permeability decreased from the unmodified membrane to Mod-ODTS and further with the addition of MOF particles, following the trend of increasing contact angle (Unmod < Mod-ODTS < Mod-F300 < Mod-ZIF-8).

To mitigate the adverse effect of hydrophobic surface properties on flux, future studies should explore optimization strategies such as introducing hydrophilic or amphiphilic surface functional groups (e.g., zwitterions, PEG-based modifiers), or implementing hierarchical surface structuring to enhance antifouling properties while preserving adsorption efficiency. Additionally, correlating roughness parameters with permeability could provide further insight into structure–property relationships. Such approaches aim to achieve a more favorable balance between selective adsorption and hydraulic performance.

In summary, based on the analysis of membrane appearance, FT-IR results, contact angle measurements, and water permeability data, it can be conclusively stated that the applied membrane modifications were effective.

### 3.7. Evaluation of Filtration–Adsorption Process

In the next stage of the research, tests of the integrated membrane-adsorption process were conducted. The feed used in the process, employing both unmodified and modified membranes, consisted of aqueous solutions of tetracycline or sulfadiazine, respectively. The process was conducted in a batch system, where the retentate and permeate streams were recirculated back to the feed tank. The changes in drug concentration in the feed tank were monitored.

**Figure 9:**
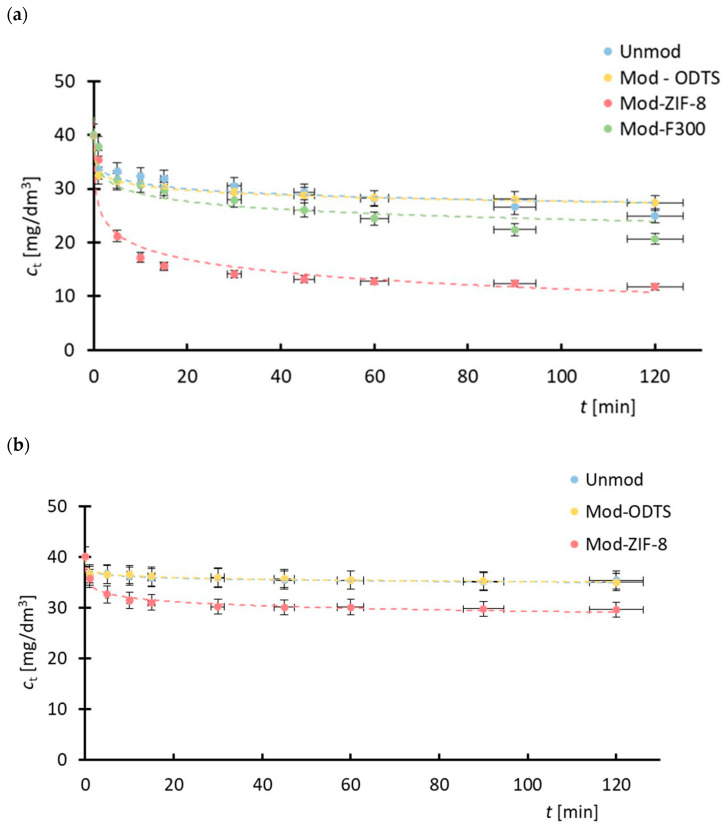
Concentration change of antibiotics during filtration: (**a**) tetracycline, (**b**) sulfadiazine. ZIF-8-modified membranes exhibit the highest removal, especially in the early phase due to fast surface adsorption.

Based on the observed changes in drug concentration in the feed during the process (Figure 9), it can be concluded that the use of membranes modified with MOF particles enhances process efficiency. For membranes coated with ZIF-8, lower final drug concentrations were achieved for both tetracycline and sulfadiazine compared to the unmodified membrane and the membrane modified solely with ODTS. In the case of the F300-modified membrane, improved efficiency was observed only for tetracycline. The concentration profiles of the pharmaceutical substances throughout the process exhibit a characteristic pattern typical of adsorption, marked by a rapid initial decline in drug concentration, followed by a gradual approach to equilibrium between the substance concentration in the aqueous phase and the amount adsorbed onto the membrane surface.

**Figure 10:**
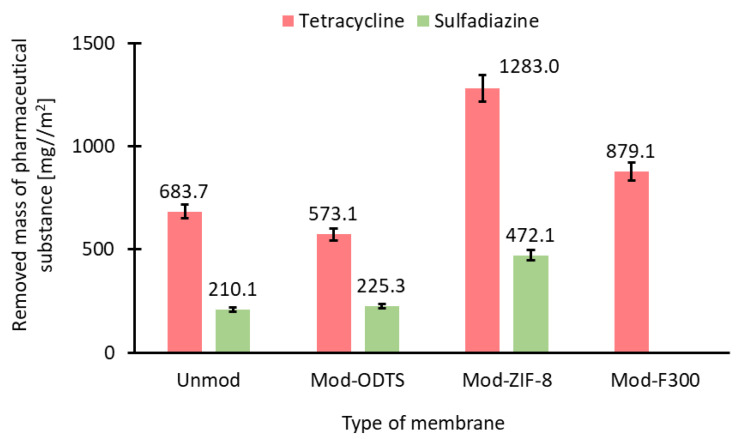
The removed mass of pharmaceutical substance for tested membrane. ZIF-8-modified membranes show the highest adsorption for both antibiotics; F300 performs well only with tetracycline.

Based on the obtained concentrations of the pharmaceutical substance, the mass of the removed pharmaceutical compound, *q_flow_* (Equation (8)), was calculated after 120 min of the process. The obtained values are presented in Figure 10. The results of *c_t_* and *q_flow_* indicate that even unmodified membranes exhibit adsorption properties toward the tested pharmaceutical substances. These properties may be attributed to the high density of -OH groups on the membrane surface, which facilitate the formation of hydrogen bonds with functional groups present in the structures of tetracycline and sulfadiazine [10]. Moreover, the abundance of -OH groups in tetracycline may explain its greater adsorption capacity compared to sulfadiazine on the surface of an unmodified membrane. Additionally, the obtained *q_flow_* values confirm the inability of F300 to adsorb sulfadiazine and demonstrate that F300 exhibits weaker adsorption properties for tetracycline compared to ZIF-8.

**Figure 11:**
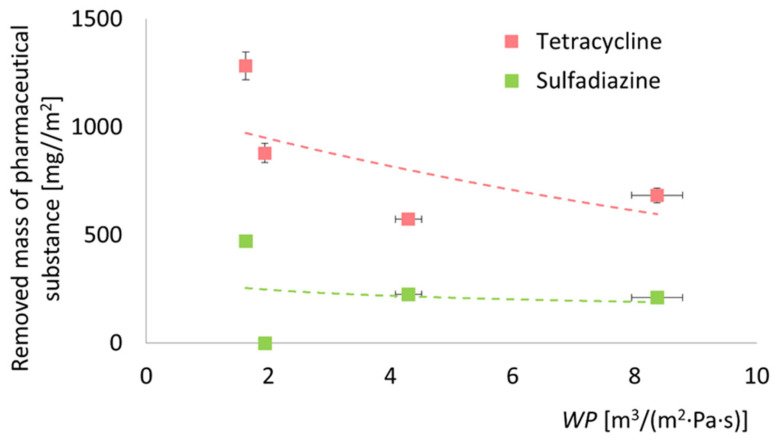
Comparison of adsorption capacity of different membranes based on water permeability coefficient. A significant decline in membrane permeability correlated with enhanced adsorption properties of the membrane. The dashed lines presented in the figure do not possess physical significance; they serve solely to illustrate the trends of change.

Based on the data presented independently in Figure 7 (water permeability) and Figure 9 (adsorption capacity), a relationship can be established between the adsorption performance of each membrane type and its water permeability coefficient—Figure 11. The trend observed is monotonically decreasing, indicating that higher adsorption capacities are associated with lower water permeability. This suggests that improvements in adsorption performance come at the expense of permeate flux. While this trade-off is not unexpected and has been noted by other researchers [111,112], it provides a valuable basis for future membrane optimization. The goal should be to enhance adsorption efficiency without significantly compromising membrane throughput, thereby achieving a more balanced and application-ready system.

**Figure 12:**
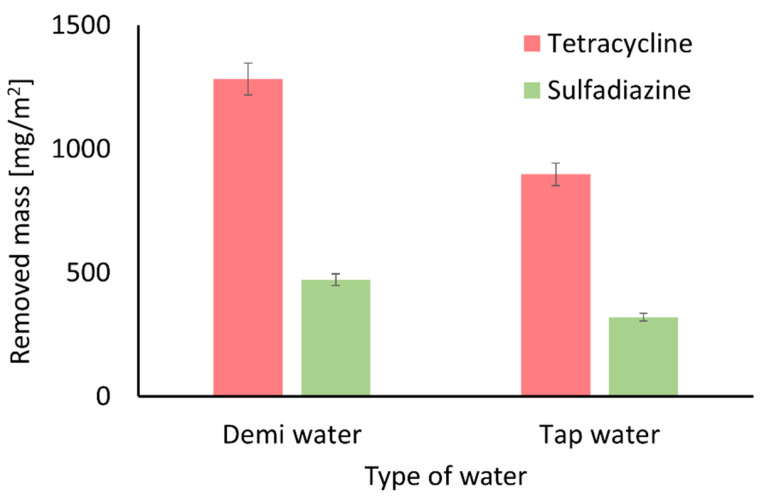
Effect of salt ions on the removed mass of tetracycline and sulfadiazine. The presence of ions in water reduces the adsorption capacity of the membranes toward antibiotics.

To investigate the influence of naturally occurring ions on the adsorption properties of the membranes, experiments were conducted using tap water as a representative matrix. Based on the results shown in Figure 12, it can be concluded that common tap water ions such as Na^+^, Mg^2^^+^, Ca^2^^+^ and Fe^2+^ reduce the amount of tetracycline and sulfadiazine removed. These findings are consistent with the literature data describing the influence of such ions on the adsorption of pharmaceuticals from water [101,113,114]. The presence of these ions weakens electrostatic interactions between antibiotic molecules and the membrane surface due to the electrostatic screening effect [115]. Additionally, positively charged ions may compete with antibiotic molecules for adsorption sites on the negatively charged surface of ZIF-8 structures.

When analyzing the results presented in this study, it is essential to compare them with similar parameters reported in other studies. Such a comparison is summarized in Table 5. It should be noted that this table includes various membrane processes. In the present study, the microfiltration process was investigated. Therefore, the most relevant references are studies on microfiltration and ultrafiltration processes.

Nanofiltration, reverse osmosis, and forward osmosis are, by nature, highly efficient in removing pharmaceutical compounds from water. Due to the specific characteristics of the membranes used in these processes and the filtration mechanisms involved, pharmaceutical retention rates exceeding 80% can be achieved [116,117,118,119,120,121], which is unattainable for microfiltration and ultrafiltration techniques. Moreover, membranes used in nanofiltration, reverse osmosis, and forward osmosis can be further modified to enhance their ability to remove pharmaceuticals from water [122,123].

In contrast, membranes used in microfiltration and ultrafiltration, which are characterized by relatively large pore sizes, exhibit low retention rates for pharmaceutical substances. The retention rates achievable in these processes primarily result from the adsorption of pharmaceutical compounds on the membrane surface. However, as confirmed by both the findings of this study and the literature data, the ability of microfiltration and ultrafiltration membranes to remove pharmaceuticals from water can be improved through surface modification [120,121,124].

Based on the data compiled in Table 5, it can be inferred that the rejection rates obtained, particularly for tetracycline, are comparable to those reported in other studies. When comparing different membrane techniques, it is important to note that microfiltration is a less energy-intensive process than ultrafiltration, nanofiltration and reverse osmosis. Additionally, the performance of microfiltration, measured as the permeate flux per unit membrane area, is higher than that of other membrane filtration processes. Therefore, despite achieving lower pharmaceutical rejection rates, microfiltration can serve as an alternative to the more commonly used high-energy and high-pressure filtration processes.

**Table 5:** Pharmaceutical removal by membrane technics—comparison.

Membrane Type/Material	Membrane Process	Pharmaceutical Substance	Rejection [%]	Reference
Al_2_O_3_ + ZIF-8	MF	**Tetracycline**	70.6	This work
		**Sulfadiazine**	25.9	
PVDF + PVP-TiO_2_-Dopamine	UF	**Sufladiazine**	~70	[125]
COF-LZU1	UF	**Tetracycline**	34.7–82.5	[126]
		**Sulfadiazine**	4.3–78	
NF-90	NF	Sulfamethoxazole	>95	[116]
		Carbamazepine	>95	
		Ibuprofen	>95	
UF ceramic TiO_2_ + GO	UF	Ibuprofen	~70	[124]
		Diclofenac	~80	
		Carbamazepine	~25	
		Naproxen	~70	
PVDF	UF	**Sulfadiazine**	48.62	[120]
PP			51.55	
NF-90	NF	Carbamazepine	bd	[118]
		Diatrizoate	98	
NF-270	NF	Carbamazepine	92	[118]
		Diatrizoate	97	
NF-90	NF	**Sulfadiazine**	~95	[127]
NF-270			~90	
PSf + GO	NF	Ibuprofen	~95	[122]
SW30	RO	Carbamazepine	~100	[118]
		Diatrizoate	~100	
TFC	FO	**Tetracycline**	~99	[119]
TFC + TiO_2_	FO	Metoprolol	~90	[123]
		Sulfamethoxazole	~100	
		Triclosan	~100	

MF—microfiltration, UF—ultrafiltration, NF—nanofiltration, RO—reverse osmosis, FO—forward osmosis.

In summary, this study proposes an innovative and practical approach to enhancing membrane performance by integrating commercially available MOFs into microfiltration systems. The modification method, based on silane-assisted MOF deposition, enables the transformation of passive ceramic membranes into active hybrid materials that simultaneously perform filtration and adsorption. This strategy offers a promising alternative to conventional high-pressure processes by combining operational simplicity, scalability, and energy efficiency with enhanced removal of pharmaceutical contaminants. By effectively bridging the gap between fundamental MOF adsorption studies and real-world membrane applications, the work lays a foundation for broader implementation of advanced materials in sustainable water treatment technologies.

### 3.8. Membrane Regeneration

The membrane regeneration process, aimed at desorbing tetracycline and sulfadiazine from the membrane surface, was conducted in a flow-through system as a subsequent stage of the filtration–adsorption operation. Regeneration was performed under continuous permeate collection at a feed pressure of 0.5 bar. The procedure comprised two distinct stages. In the first stage, the membrane was rinsed three times with ethanol, with a total volume of 0.2 dm^3^. The application of ethanol served a dual purpose: it facilitated membrane regeneration and enabled the recovery of antibiotics in the form of an organic solution. In the second stage, the membrane was rinsed with ultrapure water adjusted to pH 10, using a total volume of 1.5 dm^3^. Alkaline rinsing is a well-established method in membrane cleaning protocols [128,129]. Upon completion of the regeneration procedure, the membrane was flushed with ultrapure water and subsequently air-dried.

**Figure 13:**
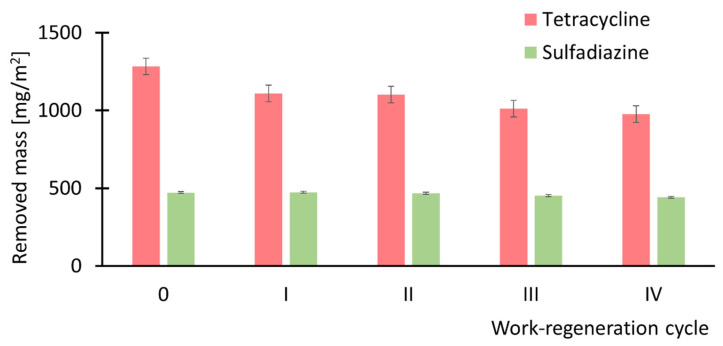
Amount of antibiotics removed by the studied membranes after successive regeneration cycles. In the case of tetracycline, a noticeable decline in adsorption performance is observed with each regeneration cycle, whereas for sulfadiazine, the adsorption properties remain largely unchanged.

Based on the results presented in Figure 10, further investigations focused on Mod-ZIF-8 membranes, which exhibited favorable adsorption properties toward both antibiotics. To evaluate membrane durability, and in particular their adsorption capacity, a series of four adsorption–regeneration cycles was conducted. In each cycle, the amount of pharmaceutical compound adsorbed onto the membrane was determined during a subsequent filtration–adsorption process. Figure 13 presents the measured mass of antibiotic adsorbed onto the membrane: initially (fresh membrane) and after each of the four regeneration cycles.

Based on the results presented in Figure 12, it can be concluded that repeated work–regeneration cycles do not significantly impair the performance of ZIF-8-modified membranes. However, a slight decline in the adsorption properties of the membrane for tetracycline is observed with successive regeneration cycles, whereas the properties regarding sulfadiazine remain practically unchanged. Furthermore, no observable changes in membrane permeability were detected throughout the cycles. Importantly, the permeability values measured after each regeneration cycle remained within the standard error range of the value obtained for the fresh membrane, i.e., 1.69 × 10^9^ m^3^/(m^2^·Pa·s)—see Figure 7, indicating that the structural integrity and surface characteristics of the modified membranes remain stable during regeneration.

During the membrane regeneration study, measurements of the pharmaceutical substance released into the desorption agent (ethanol or NaOH) were conducted after each stage of a given cycle. The results for tetracycline and sulfadiazine, respectively, are summarized in Table 6. For tetracycline, 68% of the adsorbed mass was successfully recovered, while the recovery for sulfadiazine was remarkably higher at 87%. These findings are consistent with the data presented in Figure 12.

It is also noteworthy that the efficiency of the regeneration process could be further enhanced by employing alternative organic solvents, increasing the contact time, or elevating the temperature of the regeneration solutions. Long-term experiments conducted using a single membrane additionally confirm that the applied modification strategy facilitates the development of robust and reusable membrane structures.

## 4. Conclusions

This study demonstrated the efficacy and practical potential of integrating metal–organic frameworks (MOFs), specifically ZIF-8 and F300, into membrane-based filtration–adsorption systems for the removal of pharmaceutical compounds—tetracycline and sulfadiazine—from aqueous media. The results from batch adsorption experiments revealed that ZIF-8 exhibits outstanding adsorption capabilities for both antibiotics, with maximum uptake capacities of 442.2 mg/g for tetracycline and 219.3 mg/g for sulfadiazine, significantly outperforming F300, which was effective solely for tetracycline. Kinetic modeling indicated that the adsorption process follows pseudo-second-order and Elovich models, suggesting that chemisorption and heterogeneous surface interactions are the dominant mechanisms. Furthermore, isotherm analyses confirmed that adsorption occurs predominantly via monolayer formation, with the Langmuir and Redlich–Peterson models providing the best fits.

The study also underscored the critical influence of pH on adsorption efficiency, with tetracycline achieving optimal removal at pH 6–8 and sulfadiazine at pH 4–6. These findings align with the zwitterionic and neutral forms of the respective pharmaceuticals and the surface charge characteristics of the MOFs. Membrane modifications via silane-assisted deposition of MOF particles proved successful, as verified by FT-IR spectra, contact angle measurements and water permeability analysis. ZIF-8-modified membranes attained superhydrophobic properties and exhibited the highest adsorption capacity among all tested configurations, albeit at the cost of reduced water permeability.

When incorporated into a microfiltration system, ZIF-8-modified membranes significantly enhanced antibiotic removal compared to unmodified membranes by 187% for tetracycline and 224% for sulfadiazine. Although membrane permeability decreased due to surface hydrophobization, the observed values remained within acceptable ranges for practical application. Moreover, the membranes retained structural and functional integrity over multiple adsorption–regeneration cycles, with consistent permeability and minimal loss of adsorption capacity, especially for sulfadiazine. Approximately 68% and 87% of adsorbed tetracycline and sulfadiazine, respectively, were successfully recovered through a two-stage regeneration process.

In conclusion, the hybrid membrane-adsorption approach utilizing ZIF-8 offers a scalable, energy-efficient, and cost-effective solution for removing antibiotics from water, effectively bridging the gap between laboratory-scale MOF applications and real-world water treatment demands. This work lays a solid foundation for the further development of functionalized membrane technologies tailored for environmental remediation, with potential extensions toward other classes of micropollutants.

## Figures and Tables

**Table 2:** Kinetic parameters for tested drug–MOF system.

MOF	Pharmaceutical Substance	*q*_120_ (exp) [mg/g]	Pseudo-First-Order Model			Pseudo-Second-Order Model		
			*q_e_* [mg/g]	*k*_1_ [1/min]	*R* ^2^	*q_e_* [mg/g]	*k*_2_ [mg/(g·min)]	*R* ^2^
ZIF-8	Tetracycline	442.2	405.6	0.06	0.94	472.5	32.12	0.98
	Sulfadiazine	219.3	200.5	0.83	0.87	209.5	256.88	0.95
F300	Tetracycline	170.4	149.3	0.18	0.97	158.5	62.41	0.98

**Table 3:** Parameters of the tested isotherms.

MOF	Pharmaceutical Substance	Redlich–Peterson					
		*k_R_*	*B*	*β*	*R* ^2^	*SSE*	*p*
ZIF-8	Tetracycline	13.1	0.001	0.84	0.99	908,750	0.41
	Sulfadiazine	9.4	0.027	1.15	0.98	70,711	0.41
F300	Tetracycline	7.4	0.006	0.83	1.00	86,203	0.45
		Sips					
		*k_L_*	*q_max_*	1/*n*	*R* ^2^	*SSE*	*p*
ZIF-8	Tetracycline	0.004	3145	0.99	0.99	1,899,596	0.39
	Sulfadiazine	0.023	488	1.10	0.99	192,995	0.39
F300	Tetracycline	0.014	507	0.98	0.99	86,027	0.47
		Langmuir					
		*k_L_*	*q_max_*		*R* ^2^	*SSE*	*p*
ZIF-8	Tetracycline	0.004	3065		0.99	4478	0.41
	Sulfadiazine	0.015	595		0.98	1680	0.41
F300	Tetracycline	0.014	499		1.00	112	0.45
		Freundlich					
		*k_F_*	1/*n*		*R* ^2^	*SSE*	*p*
ZIF-8	Tetracycline	21.6	0.81		0.99	895,036	0.33
	Sulfadiazine	17.4	0.69		0.97	70,602	0.33
F300	Tetracycline	18.3	0.61		0.99	83,481	0.36

**Table 4:** Contact angle values of tested membranes. The unmodified membrane is hydrophilic, while ODTS and MOF modifications significantly increase hydrophobicity. Mod-ZIF-8 reaches a superhydrophobic state.

	Type of Membrane			
	Unmod	Mod-ODTS	Mod-ZIF-8	Mod-F300
Contact angle [°]	<10	84.3	118.6	99.1

**Table 6:** Mass of recovered antibiotics during membrane regeneration process.

Desorption Agents	Desorption Agents Volume [cm^3^]	Antibiotics Amount in Desorption Agents			
		Tetracycline Concentration [mg/dm^3^]	Tetracycline Mass [g]	Sulfadiazine Concentration [mg/dm^3^]	Sulfadiazine Mass [g]
EtOH	200	12.32	2.46	13.64	2.73
	200	4.08	0.82	2.98	0.60
	200	2.36	0.47	0.36	0.07
NaOH	1500	3.87	5.80	0.73	1.10
Sum			9.55		4.50
Recovery			68%		87%

## Data Availability

The datasets used and/or analyzed during the current study are available from the corresponding author on request.
